# Pharmacological pain relief and fear of childbirth in low risk women; secondary analysis of the RAVEL study

**DOI:** 10.1186/s12884-018-1986-8

**Published:** 2018-08-25

**Authors:** Sabine L. M. Logtenberg, Corine J. Verhoeven, Katrien Oude Rengerink, Anne-Marie Sluijs, Liv M. Freeman, François G. Schellevis, Ben Willem Mol

**Affiliations:** 1 Department of Obstetrics and Gynaecology, Amsterdam UMC, Academic Medical Centre Amsterdam, Meibergdreef 9, 1105 ZA Amsterdam, The Netherlands; 2 Department of Midwifery Science, AVAG, Amsterdam Public Health research institute, Amsterdam, the Netherlands; 3 Department of Midwifery Science, AVAG, Amsterdam Public Health research institute, Amsterdam UMC, Vrije Universiteit Amsterdam, Amsterdam, The Netherlands; 4 Department of Obstetrics and Gynaecology Maxima Medical Centre, Veldhoven, The Netherlands; 5 Julius Center for Health Sciences and Primary Care, UMC Utrecht, Utrecht, the Netherlands; 6 Department of Obstetrics and Gynaecology, Leiden University Medical Centre, Leiden, The Netherlands; 7 Department of Obstetrics and Gynaecology, Ikazia Medical Centre, Rotterdam, The Netherlands; 8 Department of General Practice & Elderly Care Medicine, Amsterdam Public Health research institute, Amsterdam UMC, Vrije Universiteit Amsterdam, Amsterdam, The Netherlands; 9 Netherlands Institute for Health Services Research (NIVEL), Utrecht, The Netherlands; 10 Monash University Clayton, Clayton, Victoria Australia

**Keywords:** Epidural analgesia, Fear of childbirth, Labour pain, Pharmacological pain relief, Remifentanil-patient controlled analgesia, Wijma delivery expectancy/experience questionnaire (W-DEQ)

## Abstract

**Background:**

Fear of childbirth may reduce the womens’ pain tolerance during labour and may have impact on the mother-infant interaction. We aimed to assess (1) the association between fear of childbirth antepartum and subsequent request for pharmacological pain relief, and (2) the association between the used method of pain relief and experienced fear of childbirth as reported postpartum in low risk labouring women.

**Methods:**

Secondary analysis of the RAVEL study, a randomised controlled trial comparing remifentanil patient controlled analgesia (PCA) and epidural analgesia to relieve labour pain. The RAVEL study included 409 pregnant women at low risk for obstetric complications at 18 midwifery practices and six hospitals in The Netherlands (NTR 3687). We measured fear of childbirth antepartum and experienced fear of childbirth reported postpartum, using the Wijma Delivery Expectancy/Experience Questionnaire.

**Results:**

Women with fear of childbirth antepartum more frequently requested pain relief compared to women without fear of childbirth antepartum, but this association did not reach statistical significance (adjusted odds ratio (aOR2.0; 95% confidence interval (CI) 0.8–4.6). Women who received epidural analgesia more frequently reported fear of childbirth postpartum compared to women who did not receive epidural analgesia (aOR3.5; CI 1.5–8.2), while the association between remifentanil-PCA and fear of childbirth postpartum was not statistically significant (aOR1.7; CI 0.7–4.3).

**Conclusions:**

Women with fear of childbirth antepartum more frequently requested pain relief compared to women without fear of childbirth antepartum, but this association was not statistically significant. Women who received pharmacological pain relief more frequently reported that they had experienced fear of childbirth during labour compared to women who did not receive pain relief. Based on our data epidural analgesia with continuous infusion does not seem to be preferable over remifentanil-PCA as method of pain relief when considering fear of childbirth postpartum.

**Trial registration:**

Netherlands Trial Register 3687; Register date: 5 Nov 2012.

**Electronic supplementary material:**

The online version of this article (10.1186/s12884-018-1986-8) contains supplementary material, which is available to authorized users.

## Background

Labour pain is considered as severe pain [1]. Womens’ experiences of labour pain vary and are influenced by the physiological and psychological processes of birth and the extent to which women perceive pain [2]. Fear of childbirth, meaning pregnancy and childbirth related fear and anxiety, can lead to an increased pain perception [3, 4]. Due to its possible impact on both the mother and on the mother-infant interaction fear of childbirth has gained growing attention [4–6]. One can distinguish fear of childbirth antepartum –measured during pregnancy– from fear of childbirth postpartum, which is fear experienced during labour and measured after giving birth [7].

Previous studies have shown that women with fear of childbirth had reduced pain tolerance [4, 8]. Adams et al. found that women with fear of childbirth more often requested epidural analgesia during labour than women without fear of childbirth (45% vs 27%, *p* < 0.001) [9]. Saisto et al. found that 15% of the women with fear of childbirth postpartum, mentioned experienced frightening pain as principal cause of fear [10].

Pharmacological methods of pain relief are widely used during labour [2]. There is variation in the methods of pain relief and in the percentage of women using pain relief [2, 11–13]. Pharmacological pain relief reduces the pain experienced during labour [2, 14, 15]. However, childbirth satisfaction is not only influenced by pain and pain relief but also by other factors such as the attitude of the caregivers and involvement of the woman in decision making during labour [16]. There is a lack of robust data assessing the experiences of women who receive and who do not receive analgesia and their childbirth experience or well-being, including fear of childbirth.

In The Netherlands women at low risk for obstetric complications start labour in primary midwife-led care. In case of a request for pharmacological pain relief –with the exception of Entonox- women will be referred to hospital-based obstetric-led care and the primary care midwife is no longer involved in providing care. A referral during labour -for pharmacological pain relief or other medical reasons- could be influenced by fear of childbirth antepartum or could influence experienced fear of childbirth reported by women postpartum [5, 8–10, 17].

Both the relation between fear of childbirth antepartum and request for pain relief as well as administering pharmacological pain relief and experienced fear of childbirth reported postpartum, have rarely been studied in a low risk population. More knowledge about this topic could be used for counselling women for decisions regarding the use of pharmacological pain relief, the type of pain relief and the preferred place of birth.

The aim of our study was to assess, in low risk labouring women, the association between fear of childbirth antepartum and request for pharmacological pain relief (1). Furthermore, we assessed the association between the used method of pain relief and experienced fear of childbirth as reported postpartum (2).

## Methods

### Design

We studied the association between on the one hand fear of childbirth antepartum and a request for pharmacological pain relief (either remifentanil patient controlled analgesia (PCA) or epidural analgesia), and on the other hand the association between the method of pharmacological pain relief and fear of childbirth -experienced during labour- reported postpartum in women who participated in the RAVEL trial (NTR3687). This was a randomised equivalence trial among 409 low risk pregnant women comparing two pharmacological pain relief methods – remifentanil-PCA and epidural analgesia– in case of a request for pain relief during labour [14]. In this trial satisfaction with pain relief and pain scores were compared over the total duration of labour among low risk women randomised for remifentanil-PCA or epidural analgesia. Maternal satisfaction with labour pain scores and pain intensity scores were assessed hourly from the start of active labour until the second stage of labour in all participating women. Women receiving remifentanil-PCA were less satisfied with their pain relief than women using epidural analgesia [14].

The RAVEL study was approved by the ethics committee of the University Medical Centre Leiden and the boards of the six participating hospitals: Onze Lieve Vrouwen Gasthuis Amsterdam, VU Medical Centre Amsterdam, Academic Medical Centre Amsterdam, Lucas Andreas Hospital Amsterdam, St. Antonius hospital Nieuwegein, Diakonessen hospital Utrecht (ref. no. P10.240; 26 July 2012). Written informed consent was obtained of all participants and women younger than 18 years were not eligible.

The participants filled out the Wijma Delivery Expectancy/Experience Questionnaire ante-partum version (W-DEQ A) and gave their informed consent for the researchers to extract data from the maternity care record. These data were obtained by the participant’s primary care midwife. Six weeks postpartum participants received a reminder e-mail from the researchers to complete the W-DEQ post-partum version (W-DEQ B). Completed questionnaires were sent to the researchers by post.

### Definition and measurements of the outcome

Women were asked to complete the W-DEQ A during the third trimester of pregnancy and the W-DEQ B at six weeks postpartum, consistent with other studies. The W-DEQ A was developed to measure women’s feelings and fear before labour by means of the woman’s cognitive appraisal regarding the labour process. Similarly, the W-DEQ B was developed to measure, in retrospect, feelings and fear that women had experienced during childbirth. W-DEQ is a self-assessment scale -containing 33 items in both questionnaires- regarding childbirth (questions like, ‘How do you think you will feel in general during labour and delivery?’ in W-DEQ A or ‘How did you feel in general during labour and delivery?’ in W-DEQ B: extremely weak/not at all weak; extreme panic/not at all panicked; extreme trust/no trust at all). Answers are given on a six-point Likert scale ranging from ‘not at all’ (0) to ‘extremely’ (5), yielding a minimum score of 0 and a maximum score of 165, with higher scores reflecting a greater degree of fear of childbirth. According to the literature we classified the W-DEQ score in three categories [7, 9, 18, 19]. A score < 85 presenting women with low to moderate fear of childbirth; a score between 85 and less than 100 indicating women with an intense fear of childbirth, influencing the woman’s well-being, and women with a score of 100 or higher were defined as having a very intense, fobic fear. The internal consistency for both versions of the W-DEQ is shown to be good (Cronbach’s alpha = 0.93) [7].

### Definition and measurements of potentially confounding variables

We selected potentially confounding variables associated with fear of childbirth on the basis of the literature and one variable (obstetric complication) was added from clinical experience. Pre-existing psychological conditions – mainly anxiety and depression – could have an impact on the expectations of labour and could confound the relation between fear of childbirth and request for pain relief [7, 18–21]. To identify pre-existing psychological conditions, we measured anxiety and depression by using the Hospital Anxiety Depression Scale (HADS). Women were asked to complete the HADS questionnaire simultaneously with the W-DEQ questionnaire at both time points. The HADS is a questionnaire containing a 7-item anxiety scale and a 7-item depression scale, both of which have scores ranging from 0 to 21. The HADS is designed to measure depression and anxiety disorders among patients in a non-psychiatric clinic and is considered to be a reliable and efficient questionnaire [22–24]. A cut off score of ≥11 points on each scale was used to define the existence of anxiety and/or depression [24].

Other potentially confounding variables for either fear of childbirth antepartum and/or postpartum were young maternal age [18], low education level [18], previous first trimester loss [24], parity [5, 9, 17, 24], (previous) emergency caesarean section or operative vaginal delivery [5, 9, 10, 17], use of epidural analgesia during labour [9, 10], duration of labour (defined as the time from start active labour to birth) [9, 10], induction/augmentation of labour [8, 10, 17].

Partly from the literature, combined with clinical experience, we added obstetric complication as a potential confounder. In the RAVEL trial obstetric complications were defined as post spinal headache, postpartum haemorrhage (≥1000 ml in the 24 h after delivery or administration of blood products), uterine rupture, eclampsia, amniotic fluid embolism, myocardial infarction or admission to ICU) and/or neonatal admission to intensive care [10]. Due to, firstly the small study population and secondly, the low risk population in which we expected low rates of obstetric complications and interventions, we decided to combine these into one variable ‘obstetric intervention/complication’, with interventions including induction/augmentation of labour, assisted vaginal delivery, emergency caesarean section and obstetric complications into one variable. Additional file 1 shows the frequencies of these variables. As our study population consisted of women with a low obstetrical risk, women with a previous caesarean were excluded. For the second research question we added fear of childbirth antepartum as a potential confounder [17, 20].

### Analysis

To determine whether women who completed the W-DEQ questionnaires were representative for the total study population, we compared the baseline characteristics of women who completed the antepartum W-DEQ questionnaires with those of women who did not. The same was done for the postpartum W-DEQ questionnaires. To do so, we used the chi-square test, Mann-Whitney U test and the Student’s *t*-test. Prior to the analysis, the W-DEQ and HADS scores were examined for missing items. For the W-DEQ a maximum of two missing items was allowed, and missing items were assigned the mean score of all other items for that specific participant’s scale.

Multiple logistic regression analysis was used to determine whether there was an association between fear of childbirth antepartum and a request for pain relief (research question 1). All potentially confounding factors were included in the multiple logistic regression analysis, with a request for pain relief as the dependent variable.

To study whether the method of pain relief, remifentanil-PCA or epidural analgesia (randomly allocated), was associated with fear of childbirth reported postpartum we used multiple logistic regression analyses (research question 2). Because of the relatively small group of women with fear of childbirth reported postpartum we had to determine the most relevant potential confounders for the multiple regression analysis. We did a pre-selection with univariable logistic regression analysis. Variables with a *p-*value ≤ 0.2 were included in a stepwise multivariable logistic regression analysis using a backward selection method to determine factors most strongly associated with fear of childbirth reported postpartum [25]. All tests of significance were two-sided, with a *p* value ≤ 0.05 indicating statistical significance. Data were analyzed using SPSS (version 24; SPSS Inc., Chicago, IL).

## Results

**Fig. 1:**
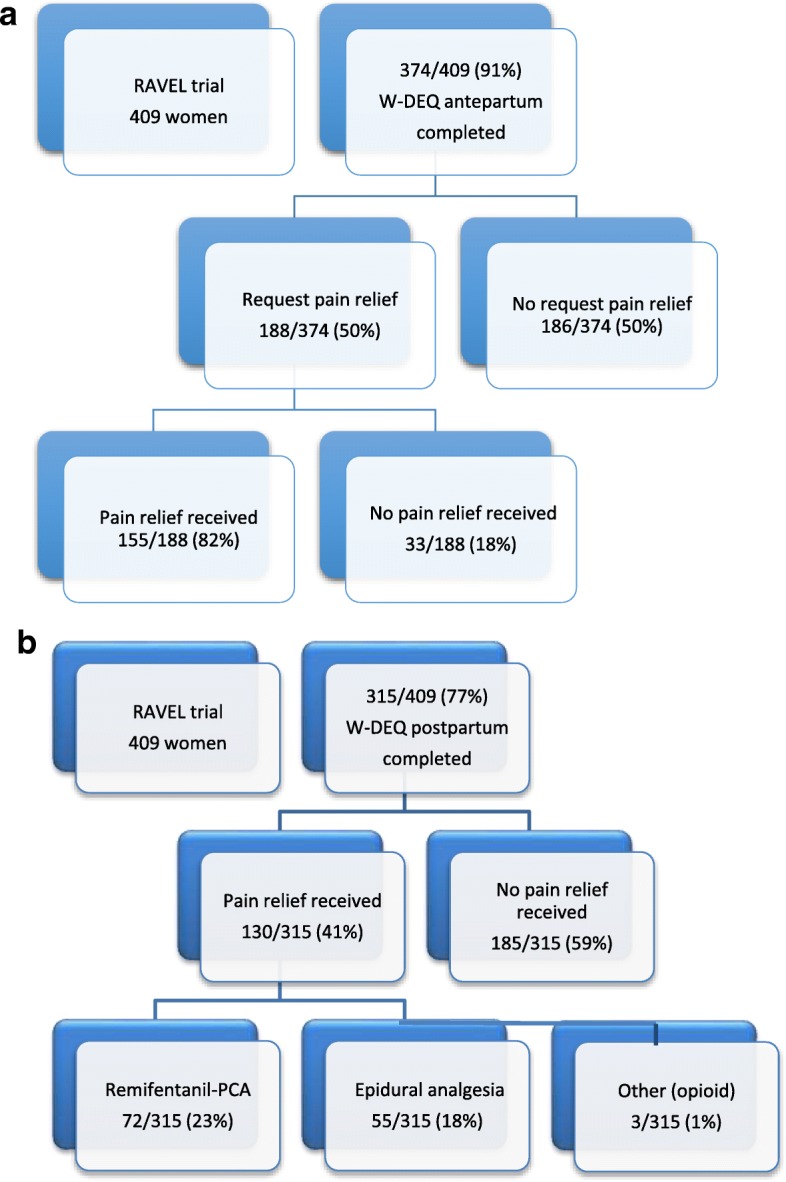
**a.** Flowchart of women in the RAVEL trial who did complete the W-DEQ A (antepartum). **b.** Flowchart of women in the RAVEL trial who did complete the W-DEQ B (postpartum)

Of the 409 women participating in the RAVEL trial, 374 (91%) women completed the W-DEQ questionnaire antepartum and 315 (77%) completed the W-DEQ questionnaire postpartum. Figure 1a&b shows the flow charts for both measures. The baseline characteristics of women who completed the W-DEQ questionnaire antepartum and women who did not were comparable. Of women who completed the W-DEQ questionnaires postpartum and women who did not, the baseline characteristics were comparable, except for the variables random allocation, ethnic origin and education level. More women randomised for remifentanil-PCA, more Western women and more women with a higher education level completed the W-DEQ questionnaires postpartum (*p* = 0.003, *p* = 0.008 and *p* = 0.004 respectively). Of the 53 women with a low education level 12 (23%) experienced (very) intense fear of childbirth postpartum compared to 24 (9%) of the 253 women with a high education level. Table 1 shows the baseline characteristics of all the women who participated in the RAVEL study and of the women who completed the W-DEQ A and the W-DEQ B.

**Table 1:** Baseline characteristics at randomisation of women who participated in the RAVEL study and who did complete the W-DEQ antepartum and/or the W-DEQ postpartum

	W-DEQ antepartum (*n* = 374) n (%)	W-DEQ postpartum (*n* = 315) n (%)	RAVEL study *n* = 409 (%)
Gestational age (weeks), median [IQR]	36 [34–38]	36 [34–38]	36 [34–38]
Maternal age (years), mean (SD)	32 (4.1)	32 (4.0)	32 (4.1)
Randomisation allocation RAVEL trial			
Remifentanil-PCA	186 (50)	169 (54)	203 (50)
Epidural analgesia	188 (50)	146 (46)	206 (50)
Ethnic origin			
Western	342 (91)	293 (93)	372 (91)
Non Western	32 (9)	22 (7)	37 (9)
Education			
≤ Low-medium professional school	75 (20)	53 (17)	81 (20)
≥ Higher professional school	288 (77)	253 (80)	314 (77)
Unknown	11 (3)	9 (3)	14 (3)
Body mass index (kg/m^2^) mean (SD)	23 (3.4)	23 (3.4)	23 (3.4)
Parity			
0	262 (70)	222 (71)	284 (69)
≥ 1	112 (30)	93 (30)	125 (31)
Pain relief requested			
No	186 (50)	157 (50)	203 (50)
Yes	188 (50)	158 (50)	206 (50)
Pain relief received			
No	219 (59)	185 (59)	239 (58)
Yes	155 (41)	130 (41)	170 (42)
*Remifentanil-PCA*	*80 (21)*	*72 (23)*	*94 (23)*
*Epidural analgesia*	*72 (19)*	*55 (18)*	*76 (19)*
*Other (opioid)*	*3 (1)*	*3 (1)*	*3 (1)*
W-DEQ score (level of fear of childbirth)			
< 85 (low-medium fear of childbirth)	342 (91)	278 (88)	–
≥ 85 and < 100 (intense fear of childbirth)	30 (8)	26 (8)	–
≥ 100 (very intense/fobic fear of childbirth)	2 (1)	11 (4)	–

*IQR* interquartile range, *SD* standard deviation

### Association between fear of childbirth antepartum and request for pain relief

The mean W-DEQ sum score antepartum was 63 (SD 16). Thirty women (30/374, 8%) had an intense fear of childbirth, while two women (< 1%) had very intense fear of childbirth. As only two women had a score of 100 or higher, we decided to combine the women with intense and very intense fear of childbirth in one group. Of the 374 women, 188 (50%) requested pain relief (Fig. 1a). No differences were found between the characteristics of women who requested pharmacological pain relief and women who did not, except for the variable parity. The group of women with a request for pain relief consist of more nulliparous women compared to group without a request for pain relief (< 0.001)(Additional file 2). We did find an adjusted odds ratio of 2.0 (CI 0.8–4.6) between fear of childbirth antepartum and request for pain relief after adjusting for parity, maternal age, education level, previous first trimester loss, antepartum HADS score and previous vaginal instrumental delivery but this association was not statistically significant (Table 2).

**Table 2:** Association between antepartum fear of childbirth and request for pharmacological pain relief

	Request pain relief, *n* = 188 n (%)	No request pain relief, *n* = 186 n (%)	OR (95% CI)	Adjusted OR (95% CI)
Fear of childbirth antepartum^a^				
Low-medium fear of childbirth (< 85) [ref]	168 (89)	174 (94)	1.7 (0.8–3.6)	2.0 (0.8–4.6)
(Very) intense fear of childbirth (≥85)	20 (11)	12 (7)		

^a^Adjusted for parity; maternal age; education level; previous first trimester loss; antepartum HADS score; previous vaginal instrumental delivery

### Association between method of pain relief and fear of childbirth reported postpartum

The mean W-DEQ sum score postpartum was 55 (SD 24), 26 of the 315 (8%) women had experienced intense fear of childbirth and 11 (4%) women had experienced very intense fear of childbirth. We decided to combine women with intense and very intense fear of childbirth for the analysis. Of the 315 women, 130 (41%) women received pain relief: 72 (23%) women received remifentanil-PCA, 55 (18%) women received epidural analgesia, 3 (1%) women received another opioid and 185 (59%) women did not receive pain relief (Fig. 1b). Of the women who requested pain relief, 28/158 (18%) women did not receive pain relief although requested, mostly due to delivery before analgesia was in place.

In the univariable logistic regression analyses we found the administration of pharmacological pain relief to be associated with fear of childbirth reported postpartum compared to deliveries without pharmacological pain relief (*p* = 0.002). Women who received epidural analgesia, more often reported fear of childbirth postpartum compared to women who did not use pain relief (OR 4.2; CI 1.9–9.6), while the association with remifentanil-PCA was not statistically significant (OR 1.8; CI 0.7–4.3). In the univariable analysis we selected the variables fear of childbirth antepartum, parity, education level, duration of labour and obstetric intervention/complication as they met the criterion *p* < 0.2. A higher level of fear of childbirth antepartum was related to a higher level of fear of childbirth reported postpartum. Nulliparity predicted a higher chance for fear of childbirth reported postpartum than multiparity. A higher education level and a longer duration of labour were predictive for fear of childbirth reported postpartum. Also, the occurrence of an obstetric intervention/complication was predictive for fear of childbirth reported postpartum (Additional file 3).

After multivariable logistic regression analysis using backward selection with the variables parity, maternal age, education level, fear level antepartum, duration of labour and obstetric intervention/complication, the association between receiving pharmacological pain relief and fear of childbirth reported postpartum remained significant (*p* = 0.02). Women who used epidural analgesia, more often reported fear of childbirth postpartum than women who did not use pain relief (OR 3.5; CI 1.5–8.2), while for remifentanil-PCA this difference was not statistically significant (OR 1.7; CI 0.7–4.3)(Table 3).

**Table 3:** Association between whether or not pharmacological pain relief was received and fear of childbirth reported postpartum: multivariable analysis

Variable	Fear level reported postpartum low-medium (< 85) *n* = 278	Fear level reported postpartum (very) intense (≥85) *n* = 37	Adjusted OR^a^ (95% CI)
Pain relief			
*No pain relief [ref]*	174 (63%)	14 (38%)	
*Remifentanil-PCA*	63 (23%)	9 (24%)	1.7 (0.7–4.3)
*Epidural analgesia*	41 (15%)	14 (38%)	3.5 (1.5–8.2)
Fear of childbirth antepartum			
*Low-medium (< 85)*	255 (92%)	29 (78%)	3.9 (1.4–10.8)
*[ref]*			
*High (≥85 and < 100)*	16 (6%)	8 (22%)	
*& severe (≥100)*			
*Missing*	7 (3%)	0	
Education level			
(professional school)			0.4 (0.2–0.9)
*≤ Medium [ref]*	41 (15%)	12 (32%)	
*≥ Higher*	229 (82%)	24 (65%)	
*Missing*	8 (3%)	1 (3%)	

^a^Adjusted for maternal age; parity; education level; duration of labour; obstetric intervention/complication; fear of childbirth antepartum (W-DEQ A)

## Discussion

### Main findings

We observed that women with fear of childbirth antepartum more often requested pain relief, although this association did not reach statistical significance. The results of our analyses also suggest that women who received pharmacological pain relief more often reported experienced fear of childbirth postpartum compared to women who did not use pain relief. This association was statistically significant for women who used epidural analgesia with continuous infusion, while it did not reach statistical significance for women who used remifentanil-PCA.

### Interpretation

The frequency of fear of childbirth antepartum (8,5%) and fear of childbirth reported postpartum (11,7%) in our study was in accordance with the literature [5, 10, 17]. Comparable with previous studies, we observed that women with fear of childbirth antepartum were more likely to request pain relief during labour, although our study did not show statistical significance [8, 10, 26]. This might be because of a lack of power in our study. Another explanation could be that our study population consisted of low risk labouring women under the care of a primary care midwife. It is assumed that women receive continuous support of labour during midwife-led care. Previous research showed that continuous support of labour results in less pharmacological pain relief [27–29]. Also, one might assume that these healthy women probably have other expectations towards pain and the use of pharmacological pain relief compared to women with a medium to high obstetric risk already under care of an obstetrician. Besides, it is known that there are different reasons for fear of childbirth antepartum, like fear of the unknown, loss of control and labour pain [5]. Geissbuehler et al. found fear of labour pain as one of the most frequent reasons for fear of childbirth [29]. However, we did not have information about the background of fear of childbirth in our study population.

Our study shows that women who used epidural analgesia with continuous infusion more often report fear of childbirth postpartum compared to women who did not use pharmacological pain relief. We did not find this relation for the use of remifentanil-PCA. Previous studies did not distinguish between the method of pain relief, but usually only reported about epidural analgesia. To our knowledge, there are no studies about the association between remifentanil-PCA and fear of childbirth. Possibly, the effect of remifentanil-PCA at the birth experience towards experienced fear of childbirth is different from the effect of epidural analgesia- a more invasive method of pain relief. Although epidural analgesia gives lower pain scores and a better satisfaction with pain relief, our study suggests that women who received remifentanil-PCA do not report more fear of childbirth postpartum compared to women who used epidural analgesia with continuous infusion [14, 15].

Our study population contains mainly Western, highly educated women. In addition, less women with a low education level completed the W-DEQ postpartum compared to women with a high education level. This may have influenced of the results of our study. Fear of childbirth reported postpartum occurred more often in women with a low education level (23%) compared to women with a high education level (9%). This is in accordance with Laursen et al. who found that fear of childbirth is expected to be higher in women with a lower education level [18]. Therefore, our results are probably an underestimation of fear of childbirth reported postpartum by low-risk pregnant women which limits the generalisability of our study.

The result that fear of childbirth antepartum is cogent related to fear of childbirth reported postpartum is consistent with previous studies [7, 17, 20, 24]. The childbirth experience is more affected by already existing antepartum fear of childbirth than by interventions or complications during labour [20, 30]. It is shown before that fear of childbirth is associated with obstetric intervention/complications as well as with caesarean section as preferred mode of delivery [5, 9]. This knowledge makes it important to distinguish fear of childbirth antepartum. Once recognized, women can make an informed choice for treatment of their fear of childbirth in order to prevent both obstetric intervention/complications and fear of childbirth reported postpartum as well as perinatal costs [30–32].

### Strengths and limitations

The main strength of our study is the high response rate for both questionnaires, antepartum (91%) and postpartum (77%). Second, our study population was extracted from the randomised RAVEL trial with many possible confounders for fear of childbirth included in the dataset. We had the opportunity to adjust for most potential confounders. Another strength is that our study distinguishes the applied method of pain relief -instead of pain relief in general- in relation to fear of childbirth reported postpartum.

Our study also has weaknesses. First, the group of women with fear of childbirth reported postpartum is relatively small. This study was a secondary analysis therefore it is possible that we did not find associations because of a lack of statistical power. Second, we combined obstetric interventions and complications into one variable ‘obstetric intervention/complication’ although the influence of every single variable at the onset of fear of childbirth postpartum could be different. Due to our small study population and the low rates of the interventions and complications it was not possible to use the individual variables in our analysis. Third, although we adjusted for the majority of potential confounding variables residual confounding could exist. Earlier research showed some other possible confounders which could influence fear of childbirth reported postpartum, for instance lack of social support, dissatisfaction with partnership and insufficient support of the caregiver [16, 21]. Information about these aspects was not available in our dataset, therefore we did not have the possibility to adjust for all potential confounders.

Furthermore, a possible weakness for the extrapolation to practice could be the population the randomised design of the RAVEL study. The fact that women knew that they were allocated to the remifentanil-PCA group or the epidural analgesia group might have influenced their psychological behaviour. For example, if women had a preference for the trial arm they were not allocated to, they would probably have tried harder to cope with labour pain without any form of labour analgesia. Besides, remifentanil-PCA and epidural analgesia were given on a request for pain relief irrespective of the stage of labour. In contrast to daily practice, when the stage of labour is taken into account to distinguish which method of pain relief will be appropriate. There could have been influence from this randomised design on the outcome measure fear of childbirth reported postpartum, for example depending of the satisfaction with pain relief the woman has experienced. In addition, the number of women who reported fear of childbirth postpartum could have been influenced by women who did not receive pain relief unless a request for it. This could have led to an overvaluation of women with fear of childbirth postpartum in the group of women who did not use pain relief. Lastly, in the RAVEL study epidural analgesia was given with continuous infusion which is associated with a greater need for provider-delivered boluses for breakthrough pain compared to patient controlled epidural analgesia. It is possible that the quality of analgesia from continuous epidural infusions negatively affected fear of childbirth postpartum [33]. To judge on extrapolation to the comparison between epidural-PCA and remifentanil-PCA further research is needed.

## Conclusions

Women with fear of childbirth antepartum more frequently requested pain relief, but this association did not reach statistical significance. Women who received pharmacological pain relief reported more frequently fear of childbirth postpartum. When looking at fear of childbirth postpartum, epidural analgesia with continuous infusion does not seem to be preferred over remifentanil-PCA as method of pain relief.

## Additional file

Additional file 1: Frequencies of the variables obstetric interventions and complications of women who completed the W-DEQ postpartum. (DOCX 13 kb)

Additional file 2: Characteristics of women who completed the W-DEQ antepartum and who did request pharmacological pain relief versus who did not. (DOCX 38 kb)

Additional file 3: Univariable analyses: association between method of pain relief and fear of childbirth reported postpartum. (DOCX 36 kb)

## Abbreviations

aOR: Adjusted Odds ratio
HADS: Hospital Anxiety Depression Scale
OR: Odds ratio
RAVEL study: Remifentanil patient controlled analgesia versus epidural analgesia study
remifentanil-PCA: Remifentanil patient controlled analgesia
W-DEQ A: Wijma Delivery Expectancy/Experience Questionnaire ante-partum version
W-DEQ B: Wijma Delivery Expectancy/Experience Questionnaire postpartum version
W-DEQ: Wijma Delivery Expectancy/Experience Questionnaire
