# Supplementary material for: Pharmacological pain relief and fear of childbirth in low risk women; secondary analysis of the RAVEL study
Source: BMC Pregnancy Childbirth. 2018 Aug 25;18:347. doi: 10.1186/s12884-018-1986-8 (PMC6109320; doi:10.1186/s12884-018-1986-8)
Supplement: Supplementary file 1 — Frequencies of the variables obstetric interventions and complications of women who completed the W-DEQ postpartum. (DOCX 13 kb) [file 12884_2018_1986_MOESM1_ESM.docx]

| **Variable*** | **N=315 (%)** |
| --- | --- |
| Induction of labour | 54 (17) |
| Augmentation of labour | 51 (16) |
| Assisted vaginal birth | 29 (9) |
| Emergency caesarean section | 31 (10) |
| Post spinal headache | 2 (1) |
| Postpartum haemorrhage | 19 (6) |
| Uterine rupture | 0 |
| Eclampsia | 0 |
| Amniotic fluid embolism | 0 |
| Myocardial infarcation | 0 |
| Maternal admission ICU | 0 |
| Neonatal admission intensive care | 0 |

*****A Woman can have more than one intervention. Rows are not mutually exclusive.

ICU: intensive care unit.
