# Supplementary material for: Pharmacological pain relief and fear of childbirth in low risk women; secondary analysis of the RAVEL study
Source: BMC Pregnancy Childbirth. 2018 Aug 25;18:347. doi: 10.1186/s12884-018-1986-8 (PMC6109320; doi:10.1186/s12884-018-1986-8)
Supplement: Supplementary file 2 — Characteristics of women who completed the W-DEQ antepartum and who did request pharmacological pain relief versus who did not. (DOCX 38 kb) [file 12884_2018_1986_MOESM2_ESM.docx]

|  |  |  | **Request pain relief**  **N=188** | **No request pain relief**  **N=186** | **P value** |
| --- | --- | --- | --- | --- | --- |
| **Gestational age** (weeks), median [IQR] |  |  | 36 [2.4] | 36 [2.3] | 0.30 |
| **Maternal age** (years), mean (SD) |  |  | 32 [4] | 32 [4] | 0.60 |
| **Randomisation allocation RAVEL trial**  Remifentanil-PCA  Epidural analgesia |  |  | 98 (52)  90 (53) | 88 (47)  98 (53) | 0.40 |
| **Ethnic origin** |  |  |  |  | 0.07 |
| Western  Non Western |  |  | 167 (89)  21 (11) | 175 (94)  11 (6) |  |
| **Education**  ≤ Low-medium professional school  ≥ Higher professional school  Unknown |  |  | 44 (23)  136 (72)  8 (4) | 31 (17)  152 (82)  3 (2) | 0.08 |
| **Body mass index** (kg/m^2^) mean (SD) |  |  | 24 [3.8] | 23 [3] | 0.10 |
| **Parity**  0  ≥1 |  |  | 153 (82)  35 (19) | 109 (59)  77 (41) | <0.001 |
| **Pain relief received**  No  Yes  *Remifentanil-PCA*  *Epidural analgesia*  *Other (opioid)* |  |  | 33 (18)  156 (82)  *80 (43)*  *72 (36)*  *3 (2)* | 186 (100) | <0.001 |
| **W-DEQ A score** (level of fear of childbirth)  <85 (low-medium fear of childbirth)  ≥85 and <100 (intense fear of childbirth) &  ≥100 (very intense/fobic fear of childbirth) |  |  | 168 (89)  20 (11) | 174 (94)  12 (7) | 0.15 |
| **HADS score antepartum**  **<** 11  ≥ 11  *missing* |  |  | 176 (96)  7 (4)  5 | 169 (92)  14 (8)  3 | 0.12 |
| **Previous first trimester loss**  No  Yes |  |  | 139 (74)  49 (26) | 139 (75)  47 (25) | 0.90 |
| **Previous assisted vaginal delivery**  No  Yes |  |  | 181 (96)  7 (4) | 173 (93)  13 (7) | 0.16 |

IQR: interquartile range; SD: standard deviation
