# Supplementary material for: Pharmacological pain relief and fear of childbirth in low risk women; secondary analysis of the RAVEL study
Source: BMC Pregnancy Childbirth. 2018 Aug 25;18:347. doi: 10.1186/s12884-018-1986-8 (PMC6109320; doi:10.1186/s12884-018-1986-8)
Supplement: Supplementary file 3 — Univariable analyses: association between method of pain relief and fear of childbirth reported postpartum. (DOCX 36 kb) [file 12884_2018_1986_MOESM3_ESM.docx]

| **Variable** | **Fear level postpartum:**  **no fear of childbirth (<85)**  **N=278; n (%)** | **Fear level postpartum: (very) intense fear of childbirth (≥85)**  **N=37; n (%)** | **OR (95% CI)** | ***p*-value** |
| --- | --- | --- | --- | --- |
| **Pain relief**  None* [reference]  RPCA or EA  *RPCA*  *Epidural* | 174 (63%)  104 (37%)  *63 (22%)*  *41 (15%)* | 14 (38%)  23 (62%)  *9 (24%)*  *14 (38%)* | 2.8 (1.4-5.6)  *1.8 (0.7-4.3)*  *4.2 (1.9-9.6)* | *0.002*  0.005  *0.20*  *0.001* |
| **Parity**  Nulli [reference]  Multi | 190 (68%)  88 (32%) | 32 (87%)  5 (14%) | 0.34 (0.1-0.9) | 0.03 |
| **Maternal age** (years) |  |  | 0.93 (0.9-1.0) | 0.09 |
| **Education level** (professional school)  ≤ medium [reference]  ≥higher  *Missing* | 41 (15%)  229 (85%)  8 | 12 (33%)  24 (67%)  1 | 0.36 (0.2-0.8) | 0.009 |
| **Duration of labour** (active labour-birth)  **Median** (hours)  *Missing* | 270  8 | 31  6 | 1.1 (1.0-1.2) | 0.007 |
| **Obstetric intervention/complication****  No [reference]  Yes | 161 (58%)  117 (42%) | 13 (35%)  24 (65%) | 2.5 (1.2-5.2) | 0.01 |
| **Hospital Anxiety Depression Scale antepartum (depression and/or anxiety)**  HADS <11 [reference]  HADS ≥11  *Missing* | 259 (96%)  10 (4%)  9 | 33 (92%)  3 (8%)  1 | 2.4 (0.6-9.0) | 0.21 |
| **Fear level antepartum**  Low-medium (<85) [reference]  High (≥85 and <100) & severe (≥100)  *Missing* | 255 (94%)  16 (6%)  7 | 29 (78%)  8 (22%)  0 | 4.4 (1.7-11.2) | 0.002 |
